# Three rhythms in one presentation: atrial flutter, torsades de pointes, and complete heart block

**DOI:** 10.1093/ehjcr/ytag665

**Published:** 2026-09-07

**Authors:** Nithin George

**Affiliations:** Department of Cardiology, Lyell McEwin Hospital, Elizabeth Vale, Adelaide, SA 5112, Australia

## Clinical vignette

**Fig. 1.**
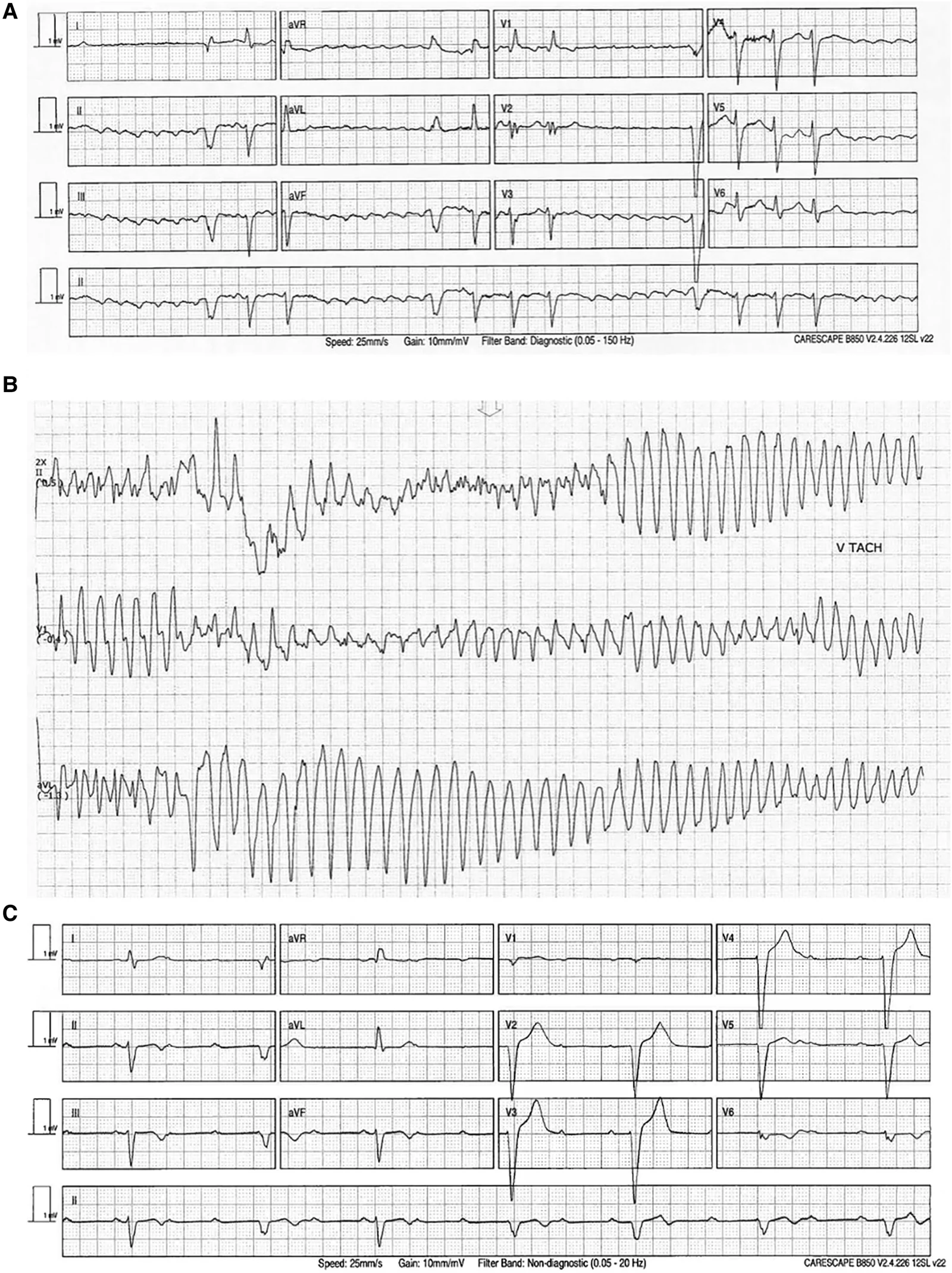
Multiple panel image (*A*) 12 lead ECG demonstrating atrial flutter with slow ventricular response consistent with tachy-brady syndrome. (*B*) Continuous ECG monitor strip (leads II, V1, aVL) demonstrating polymorphic Torsades de Pointes. (*C*) Post-ROSC ECG demonstrating high-degree atrioventricular block with wide QRS escape rhythm.

A man in his 80’s presented with 6 weeks of progressive exertional dyspnoea and bilateral lower-limb oedema, without prior syncope or known structural heart disease. His admission electrocardiogram (*Figure 1A*) demonstrated atrial flutter with a slow, irregular ventricular response (rates as low as 35 bpm). During telemetry, a late-coupled ventricular ectopic followed a prolonged RR interval and initiated a polymorphic broad-complex tachycardia with a twisting axis around the baseline (*Figure 1B*) in keeping with Torsades de Pointes. No QT-prolonging drugs or electrolyte disturbance were identified.

He was cardioverted with a single 200 J synchronized shock. Immediately afterwards the ECG showed sinus tachycardia with a markedly prolonged PR interval, atrioventricular dissociation, and a slow, wide-QRS escape rhythm consistent with complete heart block.

### Question 1

**What is the most likely mechanism of the polymorphic ventricular tachycardia shown in**  *Figure 1B***?**

- Acquired long-QT syndrome from QT-prolonging medication
- Short-coupled idiopathic ventricular fibrillation (R-on-T)
- Pause-dependent Torsades de Pointes from a short–long–short sequence
- Catecholaminergic polymorphic ventricular tachycardia
- Monomorphic ventricular tachycardia from myocardial scar

**Correct answer: C**

**Explanation:** The initiating beat is a late-coupled ectopic that follows a long, post-ectopic pause, the classic short–long–short sequence. The compensatory pause prolongs repolarisation and provokes early after-depolarisations, producing pause-dependent (bradycardia-related) Torsades de Pointes rather than the adrenergic or R-on-T mechanisms of the other options. No QT-prolonging drugs or electrolyte abnormalities were present, and the resting QT normalized at faster rates.^1^

### Question 2

**Which is the most appropriate acute management of the arrhythmia in**  *Figure 1B***?**

- Intravenous beta-blockade
- Intravenous magnesium with temporary pacing or isoprenaline to increase heart rate
- Intravenous sotalol
- Loading with intravenous amiodarone
- Digoxin for ventricular rate control of the flutter

**Correct answer: B**

**Explanation**: Pause-dependent Torsades is treated by shortening repolarisation through rate acceleration and intravenous magnesium plus temporary transvenous pacing or isoprenaline to a target rate of ∼90–110 bpm, abolishing the pauses that trigger the arrhythmia. Beta-blockade and rate-limiting agents worsen bradycardia and are contraindicated. The 2022 ESC ventricular-arrhythmia guidelines recommend magnesium and pacing/isoprenaline for bradycardia-dependent Torsades.^2^

### Question 3

**The rhythm in**  *Figure 1C*  **seen after cardioversion is best explained and managed by:**

- Reversible AV-nodal block—observation alone
- Vagally-mediated block—atropine and discharge
- Infra-Hisian conduction disease requiring permanent pacing
- Sedation-induced AV block—no further action
- Functional block secondary to the atrial flutter

**Correct answer: C**

**Explanation**: A wide-QRS escape rhythm localizes the block below the His bundle (infra-Hisian), which is unreliable and unresponsive to atropine. Per the 2021 ESC pacing guidelines, acquired complete heart block that is not due to a reversible cause is a Class I indication for permanent pacemaker implantation, in this case also correcting the bradycardic substrate for Torsades.^3^

## Lead author biography


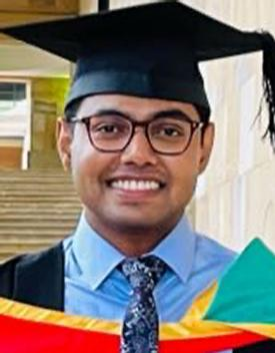


Dr Nithin George is a Basic Physician Trainee based in Adelaide, South Australia, with a strong clinical interest in cardiology and internal medicine. He holds a Master of Medicine (Internal Medicine) with a focus on cardiology and clinical epidemiology. Dr George is actively involved in research and medical education for junior doctors.

## Data Availability

Data from this case report are available on reasonable request. Please contact the corresponding author.
